# The Centennial Addresses on the Law of Similars

**Published:** 1896-04

**Authors:** Pemberton Dudley

**Affiliations:** President, A. I. H.


### THE CENTENNIAL ADDRESSES ON THE LAW OF SIMILARS.

The three addresses on the Law of Similars, provided by order of the American Institute of Homœopathy for the Centennial Celebration at Detroit next June, will be delivered as follows:

1. The Logical Basis of the Law of Similars: Does it Commend Itself to our Reason? By Richard N. Foster, M. D., of Chicago, Ill.

2. The Experimental Demonstration of the Law of Similars.

Can its Existence and Operation be Proved? By M. W. Van Denburg, M. D., of Fort Edward, N. Y.

3. The Clinical Efficacy and Superiority of the Law of Similars: Is it a Reliable Guide in the Practice of Medicine? By John P. Sutherland, M. D., of Boston, Mass.

These three addresses are designed to include and constitute a Re-examination of the Basis and Ground-work of Homœopathy, instituted after a hundred years of experimental probation and in the light of modern knowledge. They will be of a rigidly scientific character and will present, not a mere mechanical recital of facts and statistics, but a philosophic review and discussion of the subjects treated, and will be absolutely free from undignified statements and uncourteous allusions. They will undoubtedly form one of the most attractive features of the Detroit meeting.

PEMBERTON DUDLEY, M. D.,

*President, A. I. H.*

